# Prenatal Recurrence of Ductal Plate Malformations Leads to *PKHD1* Variant Reclassification

**DOI:** 10.1002/pd.6896

**Published:** 2025-10-03

**Authors:** Mario Abaji, Laurent Nasca, Marie‐Pierre Audrezet, Bénédicte Gerard, Xavier Vanhoye, Cécile Chau, Claude D’Ercole, Annie Levy‐Mozziconacci

**Affiliations:** ^1^ Centre Pluridisciplinaire de Diagnostic Prenatal Hôpital Nord AP‐HM Marseille France; ^2^ Aix Marseille Univ INSERM MMG Marseille France; ^3^ University Brest INSERM UMR 1078 GGB CHU Brest Molecular Genetics Brest France; ^4^ Eurofins Biomnis Lyon France

**Keywords:** Caroli disease, ductal plate malformation, *PKHD1*, prenatal diagnosis, variant reclassification

## Abstract

Ductal plate malformations (DPM) encompass a spectrum of congenital liver disorders characterized by abnormal bile duct development, often associated with conditions such as Caroli disease. Variants in the *PKHD1* gene cause a wide spectrum of DPM, but genotype–phenotype correlations remain challenging. We report a couple with two consecutive terminated pregnancies following prenatal detection of hepatic anomalies suggestive of DPM. Genetic analyses revealed compound heterozygous variants in *PKHD1* in both fetuses. One variant (c.931A>G) was classified as likely pathogenic, while the second (c.533T>A), initially reported as a variant of uncertain significance, was reclassified as likely pathogenic after recurrence of the phenotype. This case highlights the importance of integrating prenatal imaging, postmortem examination, and whole‐gene sequencing to refine variant classification and improve genetic counseling. Furthermore, it expands the clinical spectrum of *PKHD1*‐related disorders.

## Summary

- What's already known about this topic?
  ◦ Ductal plate malformations (DPM) encompass a spectrum of congenital liver disorders characterized by abnormal bile duct development, often linked to conditions such as Caroli disease.
  ◦ Variants in the *PKHD1* gene cause a wide spectrum of ductal plate malformations
  ◦ Prenatal imaging can detect features of DPM; however, genotype–phenotype correlations remain challenging due to limited evidence for many genetic variants.
- What does this study add?
  ◦ This study documents a case of two consecutive pregnancies with recurrent DPM, which led to the reclassification of a *PKHD1* variant (c.533T>A) from uncertain significance to likely pathogenic.
  ◦ It underscores the importance of integrating prenatal imaging, postmortem examination and whole gene sequencing to refine variant classification and enhance genetic counseling.
  ◦ Moreover, it expands the clinical spectrum of *PKHD1*‐related disorders

## 1 Fetal Phenotype

A couple was referred to the prenatal diagnosis center, following the detection of ultrasound anomalies in two consecutive pregnancies after an uneventful first two trimesters (Table 1A).

**TABLE 1A:** Clinical data.

Case	Parental details			Gestation at diagnosis	Phenotypes (HPO terms)	Obstetric history	Family history	Outcome
First pregnancy	Maternal	Age	34 yo	34 GA and 5 days	– Malformation of the hepatic ductal plate (HP:0006563) – Renal cortical microcysts (HP:0004734) – Ventricular septal defect (HP:0001629) – Left ventricular hypertrophy (HP:0001712) – Dysplastic tricuspid valve (HP:0030732) – Short philtrum (HP:0000322)	G1P0	No significant family history	Termination of pregnancy at 38 GA and 3 days
		Ethnicity	European					
	Paternal	Age	31 yo					
		Ethnicity	European					
Second pregnancy	Maternal	Age	38 yo	33 GA and 1 day	Malformation of the hepatic ductal plate (HP:0006563)	G2P0		Termination of pregnancy at 35 GA and 2 days
	Paternal	Age	34 yo					

### 1.1 First Pregnancy

**FIGURE 1:**
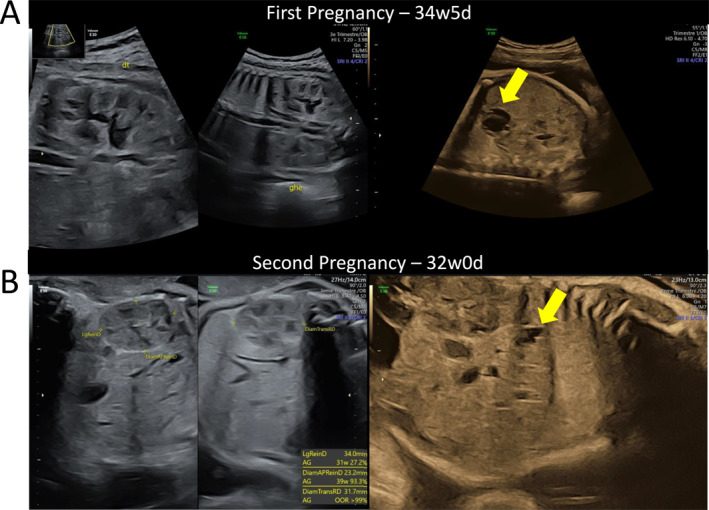
Prenatal ultrasound images from the two consecutive pregnancies showing bilateral kidneys on the left side and the liver on the right side. (A) First pregnancy at 34 + 5 GA: heterogeneous, hepatic non‐vascularized cystic‐solid mass (32 × 23 mm, arrow) (B) second pregnancy at 32 GA: multiple hepatic anechoic lesions (largest 12 × 4 mm, arrow). Kidneys increase in volume.

Routine third‐trimester ultrasound at 33 years of gestational age (GA) revealed an enlarged right adrenal gland, confirmed at the prenatal center (Figure 1A). Fetal MRI clarified the hepatic origin, identifying multiple cystic lesions, some with a tubular configuration, affecting both liver lobes. Due to the poor prognosis, the couple opted for termination of pregnancy (TOP). Postmortem examination confirmed a female fetus with a large multi‐cystic hepatic lesion, ductal plate malformation (DPM), right renal hypertrophy with possible tubular cysts, congenital heart disease and dysmorphic features.

### 1.2 Second Pregnancy

At 32 GA, ultrasound detected multiple hepatic anechoic lesions (Figure 1B), confirmed on MRI at 33 GA with a diffuse arborizing pattern and mild renal enlargement. TOP was performed and postmortem examination confirmed a female fetus with isolated DPM.

## 2 Diagnostic Method

### 2.1 First Pregnancy

Amniocentesis was performed before TOP at 38 weeks of gestation. Chromosomal microarray analysis (CMA) was normal, and targeted sequencing for hepatorenal polycystic disease genes was conducted (Supporting Information S1).

### 2.2 Second Pregnancy

Amniocentesis at 33 GA revealed a normal CMA. The same targeted analysis was performed followed by quad exome sequencing, in addition to whole gene analysis of *PKHD1* with short read sequencing (Supporting Information S1).

## 3 Diagnostic Results and Interpretation

In the first pregnancy, genetic testing using the targeted gene panel for hepatorenal polycystic disease identified two *PKHD1* variants inherited in trans. The first, c.931A>G (p.(Thr311Ala)) [1], was classified as likely pathogenic. The second, c.533T>A (p.(Val178Glu)), was initially designated as a variant of uncertain significance (VUS). After recurrence of the hepatic phenotype in the second pregnancy and identification of the same two variants, along with the exclusion of other causative variants through exome and *PKHD1* whole gene sequencing, the c.533T>A variant was reclassified as likely pathogenic (Table 1B).

**TABLE 1B:** Genetic findings.

	Variant	Zygosity	Initial criteria applied	Initial ACMG classify‐cation	Added criteria following the recurrence	Final classification
Paternal allele	NM_138694.4:c.931A>G p.(Thr311Ala)	Heterozygote	PM2_moderate, PP3_supporting, PP4_moderate, PP5_supporting, PM5_moderate	Likely pathogenic (class IV)	PP1_ supporting	Likely pathogenic (class IV)
Maternal allelle	NM_138694.4:c.533T>A p.(Val178Glu)	Heterozygote	PM2_moderate, PM3_moderate, PP4_supporting	Uncertain significance (class III)	PP1_supporting PP2_Absence of other variation detected by genome sequencing	Likely pathogenic (class IV)

## 4 Discussion

Ductal plate malformations (DPM) encompass a spectrum of congenital liver disorders including Caroli disease, a non‐obstructive dilatation of the intrahepatic bile ducts. Variants in *PKHD1*, primarily associated with autosomal recessive polycystic kidney disease (ARPKD), can also lead to a broad range of phenotypes, from asymptomatic cases to severe liver and/or kidney involvement with perinatal lethality. This phenotypic variability complicates genotype‐phenotype correlations and prenatal diagnosis [2, 3, 4, 5].

In this report, two consecutive pregnancies were terminated due to DPM. Genetic analysis revealed compound heterozygous *PKHD1* variants in both fetuses. Initially, one variant was classified as likely pathogenic and the other as a variant of uncertain significance (VUS). Reclassification of the second variant was supported by: (i) recurrence of the phenotype within the family; (ii) the high clinical specificity associated with *PKHD1* and the absence of known genetic heterogeneity for this condition; and (iii) genomic short‐read sequencing of the gene, which did not reveal any additional variants. Although the absence of additional findings does not definitively exclude complex variations that may not be detectable by short‐read sequencing (e.g., repeat expansions, large insertions/deletions, or variants in low‐mappability regions), it significantly lowers the likelihood of another pathogenic variant. This absence of additional variation may be considered an additional supporting criterion in the ACMG framework when interpreting recessive variants in genes with strong phenotype specificity.

Although congenital heart malformations have not been previously described in association with *PKHD1* mutations, children with ARPKD frequently exhibit cardiac abnormalities, including left ventricular geometric alterations and systolic dysfunction [6]. This case expands the spectrum of PKHD1‐related disorders. Thanks to this work, non‐invasive prenatal testing for the paternal *PKHD1* variant was performed in the subsequent pregnancy (Supporting Information S2: pedigree chart), confirming that the fetus was not a carrier and resulting in the birth of a healthy child.

## Ethics Statement

This study was approved by the Ethics and Scientific Committee (Comité d’Éthique et Scientifique, CSE) of AP‐HM under approval number CSE25‐95. The committee reviewed the project on March 12, 2025, and issued a favorable opinion after evaluating its scientific rationale, feasibility, patient information process, and ethical considerations.

## Consent

The couple consented for publication.

## Conflicts of Interest

The authors declare no conflicts of interest.

## Supporting information


Supporting Information S1

Supporting Information S1

## Data Availability

Data are available on request from the authors.
