# Supplementary material for: Prenatal Recurrence of Ductal Plate Malformations Leads to PKHD1 Variant Reclassification
Source: Prenat Diagn. 2025 Oct 3;45(12):1671–4. doi: 10.1002/pd.6896 (PMC12611536; doi:10.1002/pd.6896)
Supplement: Supplementary file 1 — Supporting Information S1 [file PD-45-1671-s001.docx]

**Supplementary Material**

**Methods**

*First Pregnancy :* Amniocentesis was performed before TOP at 38 GA. Chromosomal microarray analysis (CMA) was normal, and a targeted gene panel for hepato-renal polycystic disease was conducted on fœtal DNA extracted from cultivated cells searching for variants in genes involved in polycystic kidney disease after postmortem examination results. This targeted analysis was performed at Brest University Hospital using the DesignSeqCap EZ Choice Nimblegen kit (exons ±50 bp) (OID44364_v1). The analysis focused on the following genes: PKD1 (NM_001009944.3), PKD2 (NM_000297.4), GANAB (NM_198335.4), DNAJB11 (NM_016306.6), PKHD1 (NM_138694.4), HNF1B (NM_000458.4), ALG8 (NM_024079.5), ALG9 (NM_024740.2), SEC63 (NM_007214.5), PRKCSH (NM_002743.2), and UMOD (NM_003361.4). Sequencing was conducted on the Illumina MiniSeq platform. The sequences were aligned and compared to the human reference genome (GRCh37/hg19). Data analysis was performed using SeqNext v4 (JSI) and SeqOne software. In accordance with the American College of Medical Genetics and Genomics (ACMG) guidelines, only pathogenic or likely pathogenic variants (ACMG classes V and IV) were included in the reports. Variants of uncertain significance (class III) were reported only if identified as relevant (e.g., family segregation studies, functional testing). Any pathogenic or likely pathogenic variant identified in a sample was confirmed by PCR sequencing (BTDv1.1, ABI3130), and the obtained sequence was compared to the reference sequence using SeqPilot v3 (JSI) software.

*Second pregnancy*: Amniocentesis was performed at 33 GA. Chromosomal microarray analysis (CMA) was normal, and a targeted gene panel for hepato-renal polycystic disease was conducted on fœtal DNA extracted from non-cultivated cells . Quad exome sequencing was analyzed at Eurofins Biomnis laboratory using massive parallel sequencing with the Human Exome 2.0 Plus Comprehensive Exome kit, which enriches coding exons, intron/exon junctions, mitochondrial DNA, and specific intronic regions. Sequencing was performed on the Illumina NOVASEQ 6000 platform, generating 150-base paired-end reads. Quality control was ensured using 15 polymorphic markers (AS-PCR). Variants (SNVs and indels) were identified and annotated using an external pipeline (SeqOne, Hg19), while CNV analysis was conducted using both SeqOne and local pipelines. Variant interpretation followed ACMG and NGS-Diag group guidelines, with final classification validated in clinical-biological review meetings. Sequencing of the entire *PKHD1* gene (introns and exons) was performed using short read sequencing.

Applying the scoring system of Biesecker et al. (2024), segregation analysis (PP1) contributed **+5.2 points** (+4.0 for two affected fetuses, +1.2 for three unaffected individuals: both heterozygous parents and one healthy sibling). Phenotype specificity (PP4) contributed **+5 points**, given the specificity of ductal plate malformation for PKHD1-related disease. Together, these scores support upgrading the PKHD1 c.533T>A (p.Val178Glu) variant from VUS to **Likely Pathogenic**.
