# Supplementary material for: Prenatal Recurrence of Ductal Plate Malformations Leads to PKHD1 Variant Reclassification
Source: Prenat Diagn. 2025 Oct 3;45(12):1671–4. doi: 10.1002/pd.6896 (PMC12611536; doi:10.1002/pd.6896)
Supplement: Supplementary file 2 — Supporting Information S1 [file PD-45-1671-s002.pdf]

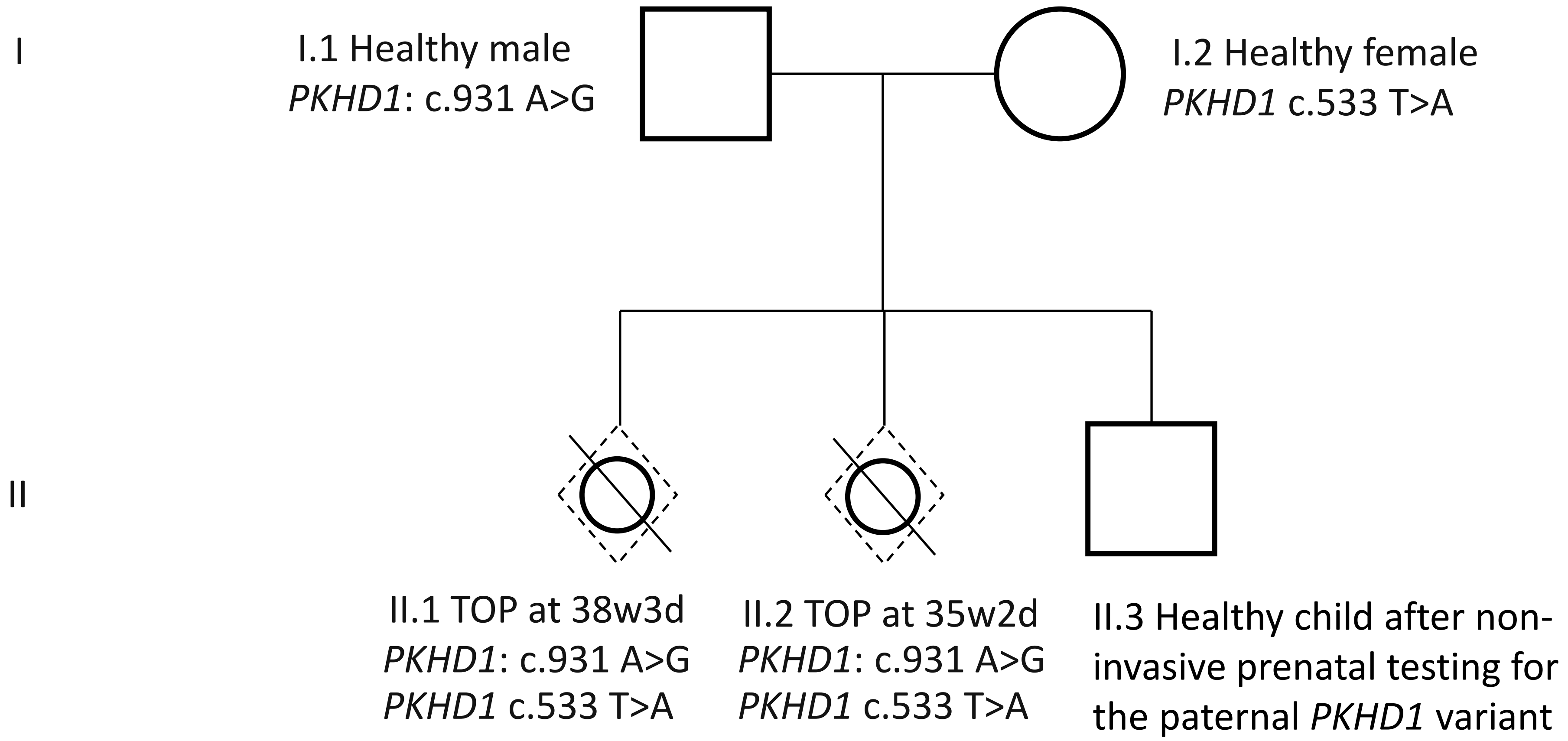

Pedigree chart: Diagram showing family individuals with their corresponding genotype, phenotype, and pregnancy outcomes.**TOP:**Termination of pregnancy.
